# Distinguishing genetically between the germlines of male monozygotic twins

**DOI:** 10.1371/journal.pgen.1007756

**Published:** 2018-12-20

**Authors:** Michael Krawczak, Bruce Budowle, Jacqueline Weber-Lehmann, Burkhard Rolf

**Affiliations:** 1 Institute of Medical Informatics and Statistics, Kiel University, Kiel, Germany; 2 Center for Human Identification, University of North Texas Health Science Center, Fort Worth, TX, United States of America; 3 Eurofins Genomics and Forensics Campus, Ebersberg, Germany; University of Washington in Seattle, UNITED STATES

## Abstract

Identification of the potential donor(s) of human germline-derived cells is an issue in many criminal investigations and in paternity testing. The experimental and statistical methodology necessary to work up such cases is well established but may be more challenging if monozygotic (MZ) twins are involved. Then, elaborate genome-wide searches are required for the detection of early somatic mutations that distinguish the cell sample and its donor from the other twin, usually relying upon reference material other than semen (e.g. saliva). The first such cases, involving either criminal sexual offenses or paternity disputes, have been processed successfully by Eurofins Genomics and Forensics Campus. However, when presenting the experimental results in court, common forensic genetic practice requires that the residual uncertainty about donorship is quantified in the form of a likelihood ratio (LR). Hence, we developed a general mathematical framework for LR calculation, presented herein, which allows quantification of the evidence in favour of the true donor in the respective cases, based upon observed DNA sequencing read counts.

## Author summary

In many instances of practical forensic casework, particularly when connected to sexual assault, genetic analysis is carried out to identify the likely donor of a sperm sample left at the crime scene. The experimental and statistical methodology for such investigations is well established. In cases involving monozygotic (MZ) twin suspects, however, the procedure is hampered by the fact that the two individuals usually coincide for the genetic markers tested. One way to overcome this problem is to use the latest DNA sequencing technology to undertake a genome-wide search for those few mutations that occur during early embryonic development and hence allow distinguishing between MZ twins in later life. Following this approach, the first cases of criminal sexual offense have been worked on successfully by Eurofins Genomics and Forensics Campus, leading to the identification of sperm sample donors from saliva reference samples taken from MZ twin suspects. As a matter of principle, however, the residual uncertainty of the experimental results needs to be evaluated and reported as well. Therefore, we developed a novel mathematical framework to quantify the evidential power of the genetic data in cases attempting to identify MZ twin donors, based upon comprehensive DNA sequencing. Moreover, we demonstrate that the same mathematical method can be used to resolve paternity disputes involving alleged fathers who have MZ twin brothers.

## Introduction

Estimates of the incidence of human twinning range from <8 per 1000 live births in Asia to >18 per 1000 live births in Central Africa [1]. This considerable geographic variation is mainly attributable to dizygotic (DZ) twinning and likely reflects the influence of social, environmental and genetic factors. The incidence of monozygotic (MZ) twins, by contrast, is rather constant at approximately 4 per 1000 live births world-wide [2].

MZ twins arise from a single zygote and therefore initially have the same genome, hence the layman’s term ‘identical’ twins. With every 1 in 250 males being a MZ twin, instances in which the presence of a genetic ‘clone’ can hamper forensic case work are more than a theoretical possibility. In fact, real life examples [3] include the 1999 case of a female student who was raped in Grand Rapids, MI, US. Five years later, DNA analysis led to the identification of a potential perpetrator, who happened to have a MZ twin brother, and both the likely candidate and his brother denied their involvement. In 2009, Malaysia police in Kuala Lumpur arrested MZ twin brothers, one of whom was a drug driver caught in the act. When the case came to court, however, there was reasonable doubt as to which twin was involved, and both men walked free. The ostensible indistinguishability of MZ twins has also challenged the probative value of genetic testing in the context of paternity disputes. For example, in 2007, a woman in the US gave birth to a child after she had had sex with MZ twin brothers. A DNA test identified both likely fathers with 99.9% probability but, owing to the nature of the genetic markers included, could not discriminate between the two men. In the end, one brother was ruled the biological father on the grounds of other circumstantial evidence.

The coalescence of all cellular lineages in a single fertilization event is the basis of the generally held view that MZ twins are indistinguishable genetically. However, after the twinning event (i.e., after the splitting of the original embryo), cell divisions along the lineages of one twin can be assumed to occur independently of the cell divisions in the other twin, at least regarding the acquisition of *de novo* mutations. Therefore, given the number of cell divisions during embryonic development and the size of the human genome, there is a reasonable chance that any two tissue samples taken from MZ twins after birth may differ regarding the presence or absence of one or more post-twinning genetic alterations.

The potential utility of this phenomenon for discriminating between the germlines of male MZ twins was highlighted in a previous thought experiment [4], suggesting an 83% probability that an offspring of a MZ twin carries at least one germline mutation (henceforth termed ‘variant’) that can be detected in his sperm sample, but not in that of his twin brother. This theoretical conjecture was corroborated empirically by Weber-Lehmann *et al*. [5] who carried out ultra-deep next generation DNA sequencing (NGS) and confirmatory Sanger sequencing in sperm samples of a MZ twin pair and a blood sample of a child of one of the twins. Five *de novo* single nucleotide substitutions were found (first by NGS and then confirmed by targeted Sanger sequencing) in the father and child, but not the uncle.

Given the technical feasibility afforded by NGS, it is anticipated that genetic MZ twin discrimination will become a common forensic practice. At the point when such testing is used in civil or criminal cases, however, the genetic expert will be required to quantify the evidential value of the laboratory results. The likelihood ratio (LR), which weighs the probability of the data under two alternative (mutually exclusive) hypotheses, is generally regarded as the most reasonable way to fulfil this requirement [6].

In the aftermath of the proof-of-principle report [5], Eurofins Genomics and Forensics Campus have been requested, by court order, to undertake similar analyses to distinguish between the germlines of MZ twin brothers. Such cases involve either the assignment of one twin to a sperm sample collected in connection with a criminal offense, or a paternity dispute. Under each scenario, DNA from saliva of the twins is used for genetic testing, i.e. the reference material is from a different tissue source than the forensic evidence (sperm or peripheral blood, respectively). Although this indirect approach may be less certain than a same-tissue comparison, most cases can be solved eventually because a sufficient number of discriminatory mutations are detected. As was noted above, however, reporting the experimental evidence must also include quantification of its probative value by way of calculating LRs. In the following, we describe and exemplify a newly developed mathematical approach to meet this demand.

## Results

### Presumptions

In our mathematical considerations, we presume that the intended germline discrimination is based upon NGS data from saliva or blood of the MZ twins, labelled A and B, that were generated as described in the Lehmann-Weber *et al*. report [5]. The same data reasonably will have served to identify potentially discriminating variants prior to the genetic analysis of the cells that derived from the germline of one of the twins. These latter cells will comprise either a sperm sample or the paternal genomic complement of an offspring of that twin, genotyped by targeted Sanger sequencing rather than NGS for reasons of the relatively large amount of input DNA required and current costs.

Following common practice, the evidential value of the genetic data is quantified by means of the LR of the two mutually exclusive hypotheses “the cells came from the germline of twin A” (hypothesis A) and “the cells came from the germline of twin B” (hypothesis B). Thus, the possibility that the cells derived from the germline of a third man essentially was ruled out beforehand on the basis of external evidence such as, for example, sufficiently discriminating short tandem repeat (STR) profiles.

Typically, DNA sequence analysis will reveal a number, n, of *de novo* mutations (most commonly single base pair substitutions) that are prevalent in both the germline-derived cells and the somatic cells of one twin but not, or in only very small amounts, in the somatic cells of the other twin. Other variants, particularly those found in only one sample, are not informative for germline discrimination and are therefore not considered any further. Moreover, since the rate of recurrent somatic mutation is of the order of 2.7×10^−9^ per base pair per mitosis [7], our mathematical considerations will rest on the presumption that every discriminating mutation traces back to one, and only one, molecular event during the development of the twins (and their germlines).

Finally, we will assume that all discriminating variants arose before the embryo split to give rise to the twins. Although most, if not all, discriminating variants will have arisen post-twinning in reality, this assumption is nevertheless reasonable because it is conservative in the sense that it systematically favours the alleged carrier of a discriminating variant, say twin A. Except for the remote possibility of a recurrent event in the germline of (non-carrier) twin B, postulating that the mutation occurred in twin A after twining would automatically rule out the possibility that the (variant-carrying) germline-derived cells came from twin B.

### Mathematical model

For theoretical reasons, it appears reasonable to assume that the (unknown) frequency, p_X_,_k_, of the k^th^ variant among the germ cells of twin X follows a beta distribution with parameters α_X_,_k_ and β_X_,_k_. In fact, the course of p_X,k_ during embryonic development can be viewed as a realization of Polya’s urn model, where single balls are repeatedly drawn from an urn containing black and white balls, each time followed by the return of the same ball and another ball of the same colour to the urn. The analogy works by equating the division of a variant-carrying cell with drawing a black ball. Probability theory then tells us that, with time, the distribution of the relative frequency of black balls in the urn, and hence p_X,k_, converges to a beta distribution [8].

The beta distributions employed here to characterise p_X,k_ result from Bayesian updates of a single prior, with parameters α and β, of the variant frequency at the end of the pre-twinning period. Updating is based upon the NGS read counts, v_X,k_ and w_X,k_, of the variant and wild-type alleles, respectively, in the body tissue sample of twin X, i.e. the germline frequency of the k^th^ mutation follows a beta distribution with parameters α_X_,_k_ = α+v_X,k_ and β_X_,_k_ = β+w_X,k_.

Reasonable initial settings of parameters α and β can be derived from a consideration of the branching process underlying early embryonic development. If the pre-twinning embryo has undergone a small number, m, of cell divisions, the frequency, p, at that stage of any post-fertilization mutation has expectation

$$E(p)=\frac{m}{2^{m+2}-4}$$ (1)

and variance

$$Var(p)=\left(\frac{2^{m}-1}{2^{m-1}}-\frac{m^{2}}{2^{m}-1}\right)/\left(2^{m+4}-16\right).$$ (2)

For a detailed derivation of formulas 1 and 2, see Materials and Methods.

In >98% of cases, MZ twinning occurs before the end of the first week of pregnancy, and 25% of twinning events pre-date blastocyst formation at day 5 [2]. The rate of cell division during early human embryonic development is fairly constant, amounting to approximately one cycle per day [9]. We may therefore reasonably assume that, on average, a pre-twinning embryo has undergone 0.25·(1+2+3+4+5)/5+0.75·(6+7)/2≈5.5 cell divisions. Setting m = 5.5 results in E(p) = 3.11×10^−2^ and Var(p) = 1.80×10^−3^. When these two figures are equated to the expectation, α/(α+β), and variance, (αβ)/[(1+α+β)·(α+β)^2^], of a beta distribution, we obtain α = 0.4895≈0.5 and β = 15.2510≈15.

Below, two types of scenarios requiring discrimination between the germlines of MZ twins will be considered, namely sperm sample donor identification and paternity testing. In line with our proof-of-principle report [5], the data underlying the likelihood calculations in both instances will be assumed to comprise (i) the NGS counts of the discriminating variants as obtained in the somatic tissue samples from the twins and (ii) the corresponding genotypes as determined in the germline-derived cells by targeted Sanger sequencing.

### Case scenarios

#### Sperm sample donor identification

The genetic data available to identify the donor of a sperm sample among MZ twins usually comprise one or more single base-pair substitutions, found in saliva of twin A and in the sperm sample, but not or at very low frequency in saliva of twin B. We also assume that no other somatic variants detected in either twin’s saliva sample have been found in the sperm sample.

Adopting the mathematical model outlined above, the frequency p_X,k_ of the k^th^ variant in the germline of twin X follows a beta distribution with parameters α_X_,_k_ = 0.5+ v_X,k_ and β_X_,_k_ = 15+w_X,k_, where v_X,k_ and w_X,k_ denote the NGS read counts of the variant and wild-type alleles, respectively. Our own casework revealed that, at sequencing coverage of between 75x and 125x, the read count ratio of discriminating variants ranges from 3:1 to 1:1 (wild-type *vs* variant) in one twin, and a maximum of one read of the variant allele is observed in the NGS data of the other twin (Table 1). For the sake of computational simplicity, and to remain lenient on twin A, we consider the presence of a single read of the variant allele in twin B the result of a single pre-twinning event, rather than a sequencing error or recurrent mutation, thereby adhering to the mathematical approach outlined above.

**Table 1:** Somatic NGS read counts and germline frequency of post-twinning mutations.

Twin	w_X,k_	v_X,k_	α_X,k_	β_X,k_	P(p_X,k_≥0.05)	E(p_X,k_)
A	50	25	25.5	65	>0.9999	0.2818
	50	50	50.5	65	>0.9999	0.4372
	75	25	25.5	80	>0.9999	0.2537
	75	50	50.5	80	>0.9999	0.3870
B	75	0	0.5	80	4.23×10^−3^	6.21×10^−3^
	100	0	0.5	115	6.01×10^−4^	4.33×10^−3^
	75	1	1.5	80	0.0414	0.0184
	100	1	1.5	115	8.01×10^−3^	0.01288

By conditioning the joint probability of the genetic data from all three samples on the joint probability of the NGS read counts observed for the twins, the latter probability cancels out in both the numerator and the denominator of the LR. Hence, the likelihood lik_k_(X) of hypothesis X essentially reduces to the conditional probability of observing the k^th^ variant in the Sanger sequencing data from the sperm sample, given hypothesis X is correct.

The detection limit of Sanger sequencing has been shown to be of the order of 5% [10] so that likelihood lik_k_(X) can be conservatively equated to the probability that the sperm sample frequency, and hence the germline frequency, of the k^th^ variant exceeds this threshold, i.e. lik_k_(X) = P(p_X,k_≥0.05). The latter values can be obtained by integration, for example, using the Casio online calculator (http://keisan.casio.com/exec/system/1180573225).

Whilst the likelihood of hypothesis A is consistently found to exceed 0.9999 for all NGS read count combinations assumed for variant-carrying twin A, variant detection in the sperm sample turns out to be rather improbable under alternative hypothesis B (Table 1). Thus, the likelihood of the sperm sample deriving from twin B ranges from 0.0414 to 6.01×10^−4^. Assuming equal prior odds, these results would lead to posterior odds of between 1:24 and 1:1664 in favor of hypothesis A.

#### Paternity Disputes

The second scenario involves one of an MZ twin pair as the alleged father of a child. STR-based genetic analysis cannot exclude one or the other man as the biological father, but usually only supports paternity of either of them and that the twins are monozygotic. Given that n discriminating variants are found, inheritance by the child of the k^th^ variant is tantamount to the event that a sperm cell randomly drawn from the germline of the biological father of the child carried the variant. Hence, likelihood lik_k_(X) would equal the frequency p_X,k_ of the k^th^ variant in the germline of twin X, if this frequency were known. Since no sperm sample is usually available from either twin, owing to court order restrictions (see Discussion), direct estimation is impossible and the sought-after frequencies must be obtained from the presumed beta distribution of p_X,k_ by way of integration, i.e. lik_k_(X) = E(p_X,k_) = α_X,k_/(α_X,k+_β_X,k_). With the same NGS read counts as assumed above, lik_k_(A) ranges from 0.2537 to 0.4372 whereas lik_k_(B) lies between 0.0184 and 4.33×10^-3^.The resulting LRs thus indicate that parenthood of twin A would be between 14 and 101 times more likely than parenthood of twin B (Table 1).

## Discussion

Identifying the male donor of a sample of germline-derived cells is a common issue in forensic casework, arising in paternity testing and in many criminal investigations, particularly in sexual offenses. Our own experience shows that both instances may also include an alleged donor who has a monozygotic (MZ) twin brother, so that unambiguous donor identification by genetic analysis alone appears challenging, if not impossible. With the advent of high throughput, time- and cost-efficient NGS technology, DNA sequencing of individual human genomes has become feasible and, in fact, relatively easy. Thus, distinguishing between the genomes of MZ twins on the basis of post-twinning somatic mutations is practical. This possibility has been demonstrated amply in studies targeting nuclear DNA [11, 12] or mitochondrial DNA from peripheral blood [13]. To our knowledge, however, successful discrimination between the germlines of male MZ twins has only been reported once before, namely by our group [5].

Donor identification among MZ twin brothers is complicated by the fact that many legal systems do not provide for the enforcement of semen donation. This limitation implies that the reference tissue used for the identification of potentially discriminating genetic variants (usually blood or saliva) differs from the target tissue (sperm). The opportunity for discriminating mutations to occur under this constraint is thus limited to the narrow developmental window separating the twinning event from the migration of the primordial germ cells into the yolk sac. This time period comprises ≤15 cell divisions and an intermediate population bottleneck, so that the number of discriminating mutations detectable in somatic tissue (i.e. blood) is likely to be very small. In fact, in the majority of cases that have been worked on so far, only two such mutations were observed, a number that is in very good agreement with its theoretical expectation of 1.78 derived in the thought experiment [4] that primed the proof-of-principle study [5] and the present work.

At first glance, one might assert that two discriminating variants seem inadequate because, normally, genetic trace donor identification or paternity testing requires consistent matching of genotypes of, for example, 10 or more short tandem repeat (STR) markers. In the setting considered here, those same STRs are applied first to reduce the potential candidates for comparison to essentially the MZ twins and, thus, exclusion is required of only one alternative candidate donor, not many candidates. Putting the possibility of a sample switch aside, in principle, a single discriminating variant would suffice to identify the source of the germline-derived cells in question. Owing to issues of sample and processing quality, however, the evidential power of the approach undoubtedly would be bolstered by the presence of a least two discriminating variants. Moreover, if these variants are located on different chromosomes, they can be assumed to be stochastically independent in the sense that the presence of one of the two underlying mutations in a given cell did not affect the probability of the presence of the other mutation (see below). Under this assumption, the joint likelihood of hypothesis X, given the genetic data, equals the product of the variant-specific likelihoods and the LR will reach a size sufficient for robust decision-making by the court or jury.

When reporting inclusionary results (i.e., matches or similar terminology), the genetic expert is usually required to quantify the evidential value of the data, and the LR is a generally accepted manner to do so. The underlying mathematical theory is well established for classical forensic applications but has not been developed yet for cases involving MZ twins. Therefore, we devised a formal framework for LR calculations by relating the unknown germline concentration of a genetic variant to its NGS read counts in somatic tissue, using Bayesian updating.

Our approach is based upon the assumption that all discriminating variants found in the alleged twin arose before twinning, which is highly conservative because it allows for the low-level presence of each discriminating variant in the other twin without invoking (highly improbable) recurrent mutations. Moreover, we employed one and the same prior for updating the frequency distributions in both twins. This strategy can easily be misunderstood as being anti-conservative because the high saliva frequency of a discriminating variant in the alleged twin may seem to require that the prior for the non-alleged twin is adjusted upwards. This argument is invalid because the cells constituting the two post-twinning embryos result from sampling *without* replacement, not from sampling *with* replacement. A high variant frequency in one twin therefore suggests a low variant frequency in the other. Moreover, the variant-bearing cells likely cluster spatially in the pre-twinning embryo because they emerge from the continued duplication of neighbouring cells. Therefore, the prior distribution rather should be adjusted downwards for the non-alleged twin, if anything, and adopting identical priors is conservative.

It should be noted that calculating likelihoods from updated beta priors alone implies that the LR is bound to converge to infinity with increasing sequence coverage. Formally, this represents a logical inconsistency because recurrent mutation in the germline of the non-alleged twin remains a possible explanation of the sequence data if one or more discriminating variants are rare or even lacking from his somatic tissue. For the level of sequence coverage pertinent in current real-world cases, this is not an issue because the beta-derived likelihoods are orders of magnitudes larger than the human germline mutation rate of 1.2×10^−8^ per base pair per generation [7]. Therefore, the model-based numbers clearly would dominate any more complex likelihood definition accounting for the possibility of recurrent germline mutation as well. However, this issue may be worth revisiting if and when advances in DNA sequencing technology indeed allow substantial increases of the sequence coverage.

As was noted above, discriminating variants on different chromosomes usually may be assumed to be stochastically independent. It must be emphasized in this context that the validity of this assumption is not a matter of high or low population frequency of the variants, or of high site-specific mutation rates (i.e. location of the variants in mutational hotspots); even highly probably events can be independent. Instead, stochastic independence between variants could be violated if the mutation rate during embryonic development varied between cell cycles in genome-wide fashion. One conceivable mechanism by which such temporal clustering of *de novo* mutations may arise is exposition to an exogenous mutagen. In this case, however, a higher overall prevalence of novel mutations would be expected to be detected in the twins. In the cases that we have worked on so far, however, the sequencing results did not show any indications of such an increase.

In conclusion, NGS has rendered genetic discrimination between the germlines of MZ twins a realistic option, fit for practical forensic casework. The few but important somatic mutations that arise early on during the development of twin embryos can now be identified with justifiable effort. Although the experimental work required in connection with such cases may have been relatively expensive to date, the costs of NGS are likely to decrease in the future. More so, our novel read count-based method of LR calculation provides a simple means to quantify the residual uncertainty about donorship in a highly conservative and, therefore, mutually acceptable way.

The current prevalence of MZ twin births [2] implies that, in ~1% of crime cases or paternity disputes, standard forensic DNA typing may turn out inadequate to resolve the potential donors. From now on, however, most cases implicating one or the other MZ twin can be successfully addressed genetically. Moreover, by highlighting the discriminatory power afforded by NGS in the special case of MZ twins, this and previous work [4, 5] should also invigorate use of this technology in other forensic contexts such as, for example, the hitherto cumbersome kinship analysis of distant relatives.

Whilst the validity of the statistical model underlying our work may occasionally require reconsideration, depending upon individual circumstances, it should represent a scientifically sound, simple and viable basis for the mathematical workup of practical cases. To put the approach in perspective, we refer to the famous quote from British statistician George E. P. Box: “Since all models are wrong, the scientist cannot obtain a ‘correct’ one by excessive elaboration. On the contrary, following William of Occam he should seek an economical description of natural phenomena.” [14]

## Materials and methods

### Ethics statement

The present work was motivated by the requirement to analyse genetic data generated on official order by investigating prosecutions or family courts. All individuals affected in such cases are invariably informed about the reason and scope of the analyses. Neither the genetic data nor the analysis results are disclosed to third parties. In order to avoid ethical conflict, the authors and the editors of PLoS Genetics therefore agreed that no genetic data or other details from real forensic casework are publicized in connection with the present work.

### Distribution parameters of de *novo* mutation frequency

Our mathematical derivations start out from a zygote carrying two identical alleles at the (autosomal) genetic locus of interest. During each round of cell division, the number of alleles present in the developing embryo is doubled, so that a variant arising from a *de novo* mutation during the k^th^ cell cycle has frequency 1/2^k+1^ among the 2^k+1^ homologous chromosomes present in the daughter cells. Moreover, the k^th^ cycle comprises the synthesis of 2^k+1^ nascent chromosomes, each representing a target of possible mutation.

Let p be the frequency of a variant that originated from one of the first m cell cycles. The above considerations imply that, after the m^th^ cycle,

$$P\left(p=\frac{1}{2^{k+1}}\right)=\frac{2^{k+1}}{\sum_{i=1}^{m}2^{i+1}}=\frac{2^{k}}{\sum_{i=1}^{m}2^{i}}=\frac{2^{k-1}}{2^{m}-1}$$

for 1≤k≤m. This leads to

$$E(p)=\sum_{k=1}^{m}\frac{1}{2^{k+1}}\cdot \frac{2^{k-1}}{2^{m}-1}=\frac{1}{2^{m}-1}\cdot \sum_{i=1}^{m}\frac{1}{2^{2}}=\frac{m}{2^{m+2}-4}$$

for the expected value of p, and

$$Var(p)=\sum_{k=1}^{m}\frac{1}{2^{2k+2}}\cdot \frac{2^{k-1}}{2^{m}-1}-\frac{m^{2}}{16{\left(2^{m}-1\right)}^{2}}=\sum_{k=0}^{m-1}\frac{1}{2^{k+4}}\cdot \frac{1}{2^{m}-1}-\frac{m^{2}}{16{\left(2^{m}-1\right)}^{2}}$$

$$=\frac{1}{16(2^{m}-1)}\cdot \left[\sum_{k=0}^{m-1}{\left(\frac{1}{2}\right)}^{k}-\frac{m^{2}}{2^{m}-1}\right]=\frac{1}{16(2^{m}-1)}\cdot \left[\frac{2^{m}-1}{2^{m-1}}-\frac{m^{2}}{2^{m}-1}\right]$$

for the variance of p.

## References

[1] Smits J, Monden C. Twinning across the developing world. PLoS ONE. 2011; 6: e25239 10.1371/journal.pone.0025239 21980404 PMC3182188

[2] Hall JG. Twinning. Lancet. 2003; 362: 735–743. 10.1016/S0140-6736(03)14237-7 12957099

[3] Jobling MA. Double trouble. Investig Genet. 2013; 4: 12 10.1186/2041-2223-4-12 23805891 PMC3716941

[4] Krawczak M, Cooper DN, Fändrich F, Engel W, Schmidtke J. How to distinguish genetically between an alleged father and his monozygotic twin: a thought experiment. Forensic Sci Int Genet. 2012; 6: 129–130. 10.1016/j.fsigen.2011.11.003 22177714

[5] Weber-Lehmann J, Schilling E, Gradl G, Richter DC, Wiehler J, Rolf B. Finding the needle in the haystack: differentiating "identical" twins in paternity testing and forensics by ultra-deep next generation sequencing. Forensic Sci Int Genet. 2014; 9: 42–46. 10.1016/j.fsigen.2013.10.015 24528578

[6] Caliebe A, Krawczak M. Probability and Likelihood In: Amorim A, Budowle B, editors, Handbook of Forensic Genetics: Biodiversity and Heredity in Civil and Criminal Investigation. London: Imperial College Press; 2015 pp. 61–80.

[7] Milholland B, Dong X, Zhang L, Hao X, Suh Y, Vijg J. Differences between germline and somatic mutation rates in humans and mice. Nat Commun. 2017; 8: 15183 10.1038/ncomms15183 28485371 PMC5436103

[8] Feller W. An introduction to probability theory and its applications. Volume II New York: John Wiley & Sons; 1966 p. 226.

[9] Herbert M, Wolstenholme J, Murdoch AP, Butler TJ. Mitotic activity during preimplantation development of human embryos. J Reprod Fertil. 1995; 103: 209–214. 7616491 10.1530/jrf.0.1030209

[10] Davidson CJ, Zeringer E, Champion KJ, Gauthier MP, Wang F, Boonyaratanakornkit J, et al Improving the limit of detection for Sanger sequencing: a comparison of methodologies for KRAS variant detection. Biotechniques. 2012; 53: 182–188. 10.2144/000113913 22963480

[11] Dal GM, Ergüner B, Sagiroglu MS, Yüksel B, Onat OE, Alkan C, et al Early postzygotic mutations contribute to de novo variation in a healthy monozygotic twin pair. J Med Genet. 2014; 51: 455–459. 10.1136/jmedgenet-2013-102197 24764354

[12] Li R, Montpetit A, Rousseau W, Wu SY, Greenwood CM, Spector TD, et al Somatic point mutations occurring early in development: a monozygotic twin study. J Med Genet. 2014; 51: 28–34. 10.1136/jmedgenet-2013-101712 24123875

[13] Wang Z, Zhu R, Zhang S, Bian Y, Lu D, Li C. Differentiating between monozygotic twins through next-generation mitochondrial genome sequencing. Anal Biochem. 2015; 490: 1–6. 10.1016/j.ab.2015.08.024 26327617

[14] Box GEP. Science and statistics. J Am Stat Assoc. 1976; 71: 791–799.

